# Improved Osteogenesis of Selective-Laser-Melted Titanium Alloy by Coating Strontium-Doped Phosphate With High-Efficiency Air-Plasma Treatment

**DOI:** 10.3389/fbioe.2020.00367

**Published:** 2020-05-12

**Authors:** Haiyuan Xing, Ruiyan Li, Yongjie Wei, Boda Ying, Dongdong Li, Yanguo Qin

**Affiliations:** ^1^Department of Orthopedics, The Second Hospital, Jilin University, Changchun, China; ^2^Key Laboratory of Automobile Materials of MOE, Department of Materials Science and Engineering, Jilin University, Changchun, China

**Keywords:** air-plasma treatment, strontium, calcium phosphate, biocompatibility, osteogenesis

## Abstract

Surface treatment and bioactive metal ion incorporation are effective methods for the modification of titanium alloys to be used as biomaterials. However, few studies have demonstrated the use of air-plasma treatment in orthopedic biomaterial development. Additionally, no study has performed a direct comparison between unmodified titanium alloys and air-plasma-treated alloys with respect to their biocompatibility and osteogenesis. In this study, the biological activities of unmodified titanium alloys, air-plasma-treated titanium alloys, and air-plasma-treated strontium-doped/undoped calcium phosphate (CaP) coatings were compared. The strontium-doped CaP (Sr-CaP) coating on titanium alloys were produced by selective laser melting (SLM) technology as well as micro-arc oxidation (MAO) and air-plasma treatment. The results revealed that rapid air-plasma treatment improved the biocompatibility of titanium alloys and that Sr-CaP coating together with air-plasma treatment significantly enhanced both the biocompatibility and osteogenic differentiation of bone marrow mesenchymal stem cells (BMSCs). Overall, this study demonstrated that low temperature air-plasma treatment is a fast and effective surface modification which improves the biocompatibility of titanium alloys. Additionally, air-plasma-treated Sr-CaP coatings have numerous practical applications and may provide researchers with new tools to assist in the development of orthopedic implants.

## Introduction

Titanium alloys are widely used as orthopedic implants for bone defects but the lack of osseointegration is a barrier to their application. Therefore, treatment methods to enhance the bioactivity of the material surface should be employed. Air-plasma treatment enhances the surface properties of materials and has been utilized in agricultural production, waste management, as well as surface cleaning and disinfection (Petlin et al., 2017; Messerle et al., 2018; Saberi et al., 2018; Thana et al., 2019). The main advantages of this process are thin film deposition, increased wettability, induced cross-linking, and surface activation of the material. Additionally, compared to wet chemical coating techniques, this process is more economical (Canal et al., 2004; Yoo et al., 2009; Hagiwara et al., 2013; Yoshida et al., 2013; Cheng et al., 2014; Zhang et al., 2019). Recently, air-plasma treatment has been implemented to improve the surface properties of biomaterials used in tissue engineering and drug delivery (Yoo et al., 2009; D’Sa et al., 2010; Chen et al., 2019). The key features of optimal biomaterials are as follows: surface protection, biocompatibility, antimicrobial and anti-tumor properties, as well as directed differentiation of stem/progenitor cells (Yoshida et al., 2013; Zhang et al., 2019). Utilizing air-plasma treatment for coatings has been shown to enhance the wear resistance and corrosion resistance of substrate surfaces (Fenker et al., 2002; Khorasanian et al., 2011; Tsunekawa et al., 2011; Wang et al., 2011; Hussein et al., 2017). Recently, various reports have demonstrated the enhanced biocompatibility of surfaces modified with air-plasma treatment (Liu et al., 2005; Lu et al., 2012). Jeong et al. reported that the hydrophilicity of the amine plasma-treated titanium alloy plate was increased relative to the control. They suggest that this treatment had a positive effect on biocompatibility (Jeong et al., 2019). Wong et al. (2013) reported that oxygen plasma surface modification in combination with aluminum enhanced cell adhesion and viability. In summary, previous studies demonstrated that air-plasma treatment significantly improved the biocompatibility of biomaterial surfaces in a quick and efficient manner.

However, ideal biomaterials not only require good biocompatibility but they must demonstrate suitable bioactivity. Air-plasma treatment has been shown to promote limited osteogenesis, as well as enhance cell proliferation and adhesion (Griffin et al., 2019). This means it is necessary to perform customized surface modification on the titanium alloy substrate in order to improve the bioactivity and osteogenesis of the implant surface. Coatings containing calcium phosphate (CaP), the main component of natural bone tissue, can promote osteogenic differentiation of stem/progenitor cells. Various ceramics, nanoparticles, and scaffolds developed with CaP have been shown to enhance osteogenesis (de Jonge et al., 2010; Alghamdi et al., 2014; Zhou et al., 2017; Othman et al., 2019). The osteogenic nature of CaP is attributed to its ability to regulate both the extracellular Ca^2+^/PO_4_^3–^ ions (Wu et al., 2003) and the adsorption/release of osteoinductive growth factors such as bone morphogenetic proteins (BMPs) (Begam et al., 2017). Furthermore, strontium (Sr)-doped CaP (Sr-CaP) coatings have been shown to have better osteogenic activity than undoped CaP coatings (Huang et al., 2017; Yu et al., 2017; Basu et al., 2019; Khalifehzadeh and Arami, 2019). Sr can increase osteoblast number, stimulate bone formation, reduce bone resorption rate, and reduce osteoclast activity and number (Yang et al., 2011; Raucci et al., 2015; Kargozar et al., 2017; Glenske et al., 2018). Together, these studies demonstrate that CaP coatings, especially the Sr-CaP coatings, are pro-osteogenic. Therefore, utilizing both plasma surface modification and Sr-CaP coating on the surface of the titanium alloys has the potential to improve the biocompatibility and osteogenesis, respectively.

Selective laser melting (SLM) has a high degree of forming freedom for titanium alloy. Based on computer reconstructed bone models, SLM can be utilized to produce orthopedic implant products with mechanical properties that are more compatible with original bones, making it possible for orthopedic clinical application (de Wild et al., 2013; Wang et al., 2019). Meanwhile, SLM technology has broad application prospects in high-strength titanium alloys and 4D printing (Kang and Yang, 2019; Lu et al., 2019), potentially leading the frontiers of orthopedic implants. In this work, we designed titanium alloy substrates (produced using SLM) modified with air-plasma surface treatment combined with Sr-CaP coating to simulate *in vivo* conditions. Titanium alloys were sintered and manufactured using SLM equipment. Sr ions were doped to the coating via micro-arc oxidation (MAO) and various assays were performed to evaluate the feasibility of these approaches to design orthopedic implants. These varied surface treatments and coating combinations allowed us to determine the optimal biomaterial surface to enhance biocompatibility and induce osteogenesis.

## Materials and Methods

### Materials

Titanium alloy disks (12 mm in diameter and 1 mm thick) were manufactured from a commercially available SLM device (Dimetal 280, Syndaya, China) using a laser power of 150 watts (W) and laser scanning speed of 500 mm/s. The device was equipped with a 500 W Yb:YAG fiber laser (spot size = 80 μm). All the disks were polished with abrasive papers and ultrasonically washed with acetone and distilled water before used.

### Preparation of Ceramic Coatings

The experimental settings for preparing the ceramic coatings were as follows: pulse frequency (500 Hz), duty cycle (10%), duration time (3 min), forward voltage (350 V), and negative voltage (50 V). During the reaction, the temperature of the electrolytes was maintained below 24°C using cooled water. The MAO electrolytes were divided into two groups depending on compositions: Group 1 (Sr-doped and labeled Sr-CaP): 0.085 M calcium acetate [(CH_3_COO)_2_Ca], 0.01 M β-glycerophosphate (β-GP) disodium, and 0.03 M strontium acetate [(C_2_H_3_O_2_)_2_Sr]; and Group 2 (undoped and labeled CaP): 0.085 M calcium acetate [(CH_3_COO)_2_Ca] and 0.01 M β-GP disodium. After the procedure, coatings were washed with water to remove any impurities.

### Air-Plasma Treatment

After MAO, all the samples were plasma-treated by using the active component (ion, atom, active group, photon, etc.). Air-plasma treatment was performed using a plasma surface treatment processor (PT-03-LF, Tiankechuangda Co. Ltd., China) to produce hydroxyl groups. The experimental settings were as follows: frequency (13.56 MHz), electric power (100 W), and airflow at 10 sccm at 0.05 T for 10 min. The plasma surface modification technique was applied to titanium alloy (TC4), CaP, Sr-CaP and the new surfaces were labeled TC4-p, CaP-p, and Sr-CaP-p, respectively.

### Surface Characterization

The morphology of the thin film coatings was observed using field emission scanning electron microscopy (FE-SEM, Hitachi8010, Japan). The mean elemental composition of each surface coating and the Ca:P atomic ratio of crystals with various morphologies was analyzed. This was done using an energy dispersive X-ray spectrometer (EDS) incorporated into the scanning electron microscope (SEM) under “area scanning” mode. The crystalline phase composition and structure of the bioceramic coatings was evaluated using an X-ray diffractometer (XRD, D8 DISCOVER, Germany) which had a monochromatic CuKa radiation source generated at 30 mA and 40 kV and a scanning speed of 4°/min with a scanning range between 15° and 60°.

### Cell Culture

Rabbit bone marrow stromal cells (rBMSCs) were obtained from the 28-day-old white rabbits (Jilin University, China). The rabbits were dissected, long bone limbs were isolated, and rBMSCs were harvested by extracting bone marrow cavities using previously described methods (Li et al., 2017). The rBMSCs were cultured in Dulbecco’s Modified Eagle’s medium with low glucose (Low/DMEM, Hyclone, United States), containing 10% fetal bovine serum (FBS, Gibco, United States) and 1% penicillin/streptomycin (Hyclone, United States) at 37°C in a humidified atmosphere of 95% air and 5% CO_2_. The medium was replaced every 2 days and cells at passage 4 were used in the following experiments. The animal procedures were performed in strict accordance with the laboratory animal regulations of the State Council of the People’s Republic of China (No. [2017] 676) and approved by the ethics committee of Jilin University.

### Cell Adhesion and Morphology

To assess cell adhesion, the rBMSCs were seeded on TC4, TC4-p, CaP-p, and Sr-CaP-p at a density of 1 × 10^5^ cells/ml in 24-well plates (Corning, United States). After 2 h of incubation, the number of adherent cells was quantified using the Cell Counting Kit-8 (CCK-8, Dojindo, Japan). The suspension was added into 96-well plates (Corning, United States) to facilitate detection using a microplate reader. To assess cell morphology, the rBMSCs were seeded at a density of 4 × 10^4^ cells/ml in 24-well plates. The cytoskeleton and cell nuclei were stained using the following protocol: (i) Incubate cells for 2 h; (ii) Wash samples with phosphate-buffered solution (PBS); (iii) Fix samples with 4% paraformaldehyde (Sangon, China) for 10 min at 4°C and stain with 50 mg/ml rhodamine phalloidin (Sigma, United States) for 1 h and 4,6-diamidino-2-phenylindole (DAPI, Sigma, United States) for 5 min at room temperature (25°C). All samples were washed with PBS between each step and each sample was visualized using a confocal laser scanning microscope (Olympus LX81-ZDC, Japan).

A separate group of cell-seeded samples at a density of 1 × 10^4^ cells/ml was fixed in 2.5% v/v glutaraldehyde at 4°C overnight. After washing with PBS, the fixed samples were dehydrated through an ethanol concentration gradient (30, 50, 75, 80, 90, 95, and 99.5%), critical-point dried, and finally sputtered with platinum. The samples were observed through the FE-SEM (XL-30 ESEM FEG Scanning Electron Microscope, FEI Company, United States).

### Cell Proliferation

The rBMSCs were seeded on different surfaces at a density of 4 × 10^4^ cells/ml in 24-well plates. At 1, 4, and 7 days, the samples were moved to fresh 24-well plates, respectively, before being gently washed by PBS. Cell proliferation was assessed using CCK-8 kit (CCK-8:DMEM = 10:100) according to the manufacturer’s instructions (2 h incubation at 37°C) and was quantified using optical density (OD) values at 450 nm on a microplate reader (Varioskan Flash, Thermo Scientific, United States).

### Cell Viability

The rBMSCs were seeded at a density of 2 × 10^4^ cells/ml and incubated for 1 day. Apoptosis was evaluated using Cell Viability Assays (Invitrogen, Life Technologies, Carlsbad, CA, United States) and quantification was performed using the FR-1800 luminescent and fluorescent biological image analysis system (Furi Science and Technology Co., China).

### Alkaline Phosphatase (ALP) Activity Assay

The rBMSCs were seeded on samples at a density of 6 × 10^4^ cells/ml in 24-well plates and incubated until blank group cells had mostly adhered. Subsequently, the cells were cultured with the osteogenic medium supplemented with 50 mg/l ascorbic acid, 10^–8^ M dexamethasone, and 10 mM β-glycerol phosphate (Sigma, United States). The medium was changed every 2 days. Alkaline phosphatase (ALP) staining was performed 7 days after the addition of the osteogenic medium using the BCIP/NBT ALP Color Development Kit (Beyotime, China). To quantify ALP activity, the samples were washed with PBS and cells were disrupted using RIPA lysis buffer (Beyotime, China). The cell lysate was collected for determining both the ALP and total protein concentration using ALP Assay Kit (Beyotime, China) and BCA Assay Kit (Beyotime, China), respectively. After 30 min incubation at 37°C, the ALP concentration and total protein concentration were measured using a microplate reader (Varioskan Flash, Thermo Scientific) at wavelengths of 405 and 562 nm, respectively. The ALP concentration was calculated according to a standard curve and normalized to total protein concentration while the ALP activity was normalized to incubation time. Specifically, the following equation was used to calculate ALP activity.

ALP activity (nmol/min/mg) = (ALP concentration/total protein concentration)/incubation time.

### Alizarin Red Staining

After incubation for 7 and 14 days with osteogenic medium, the samples were fixed with 4% paraformaldehyde for 10 min at 4°C and stained with alizarin red (pH = 4.2) at room temperature (25°C). Images were visualized using a zoom stereo microscope.

### Statistical Analysis

All the assays in this study were performed in triplicate. Data was presented as the mean ± standard deviation (SD). Statistical analysis was performed using multiple *t*-tests with the GraphPad Prism software. The value *p* < 0.05 was considered statistically significant.

## Results

### Characterization

**FIGURE 1:**
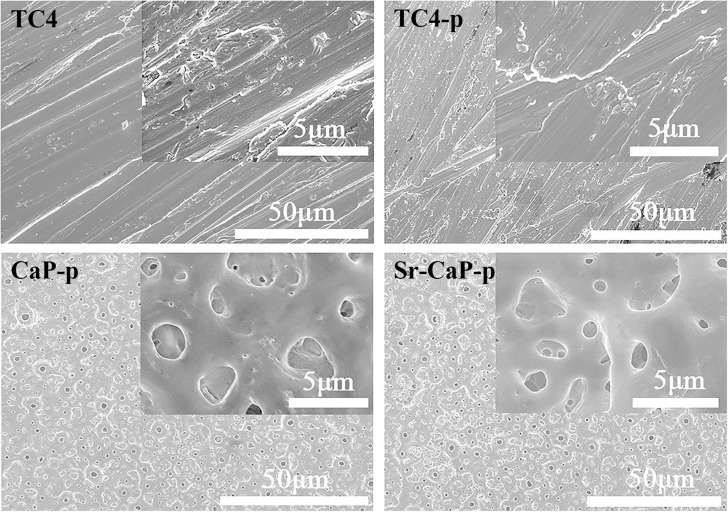
SEM images of TC4, TC4-p, CaP-p, and Sr-CaP-p.

The morphological features of the coated titanium alloy samples were characterized by SEM. Figure 1 shows SEM images of the specimen surfaces: TC4, TC4-p, CaP-p, and Sr-CaP-p. The surface morphology of TC4 exhibited an irregular shape with numerous artificial scratches due to the mechanical polishing process (Figure 1). There was no change in the topographic characteristics (morphology or average roughness values) of TC4 surfaces after plasma treatment. After MAO modification, a volcanic eruptive microporous structure was observed on the sample. The pore diameter of the coating was about 1–3 μm and distributed at regular intervals. At higher magnifications, the surface of the coating was observed to be quite smooth. The introduction of strontium ion did not change the morphology of the coatings.

The chemical composition of Sr-CaP-p was analyzed by EDS. The element mapping diagrams were consistent with the surface topography of Sr-CaP-p, suggesting that all the elements were evenly distributed (Supplementary Figure S1). The element Ti is incorporated into the coating from the substrate, and the elements Ca, P, Sr originated from the electrolyte solution. The percent by weight (wt%) of Ca and P in Sr-CaP-p was 6.17 and 4.25 wt%, respectively. The Ca:P ratio was approximately 1.12, which is close to the Ca:P stoichiometric ratio of HA at 1.67. Bioactive surface coatings containing CaP, which mimic *in vivo* bone tissue, have been widely developed for load-bearing implant applications. The introduction of Sr ions into the coatings can accelerate the formation and calcification of bone tissue, thereby reducing fracture healing time. The wt% of Sr in Sr-CaP-p is approximately 1.97 wt%.

**FIGURE 2:**
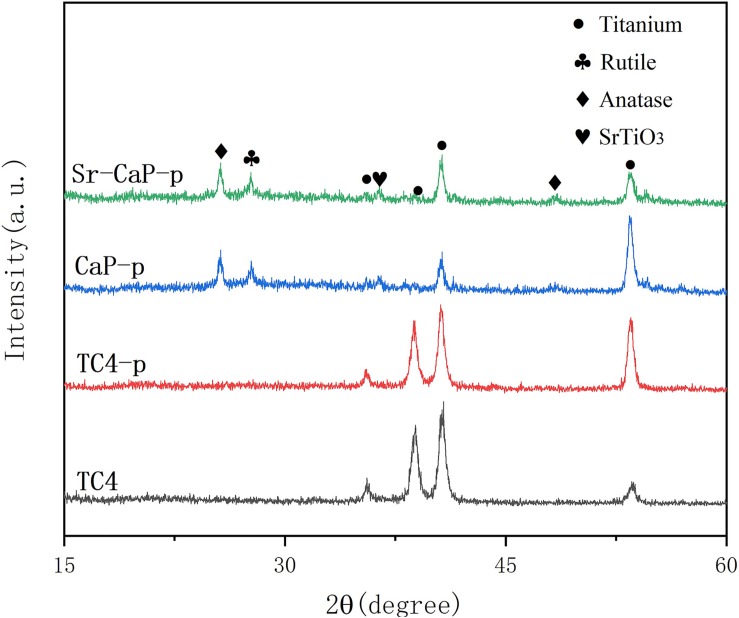
XRD spectra for TC4, TC4-p, CaP-p, and Sr-CaP-p.

The osseointegration of implants is strongly related to their surface composition (Zhang et al., 2015). The XRD patterns of the different coatings are shown in Figure 2. TC4 and TC4-p had similar peaks after plasma treatment and this suggested to us that this modification did not change the structure and composition of materials. The main substance in the coating of CaP-p and Sr-CaP-p is the diffraction peak of anatase and rutile with low crystallinity, and the diffraction peak of titanium alloy is derived from the TC4 matrix. Rutile and anatase peaks can be clearly detected due to the MAO process, which was in conformance with JCPDS 96-900-7433 and JCPDS 1-86-1156, respectively, demonstrating their potential biocompatibility.

**FIGURE 3:**
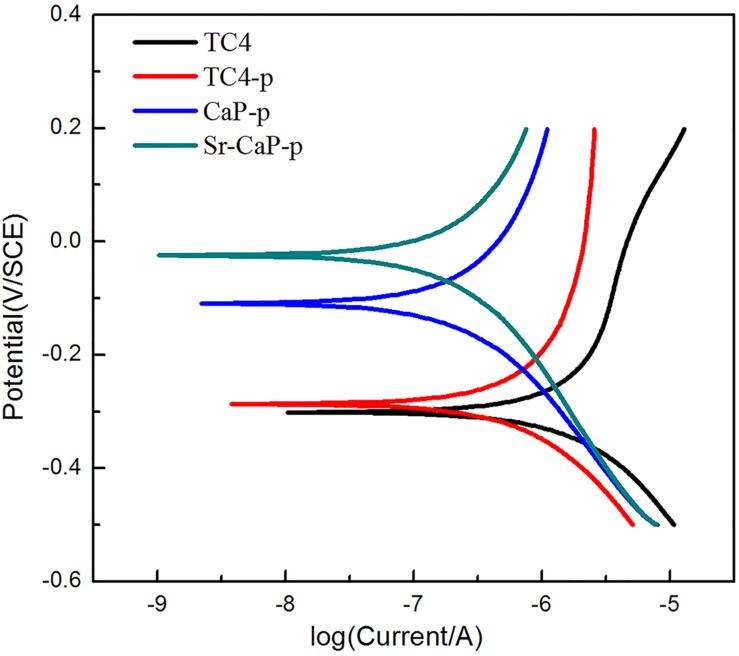
Potential dynamic polarization curves of TC4, TC4-p, CaP-p, and Sr-CaP-p.

The potentiodynamic polarization curves of TC4 before and after modification are shown in Figure 3. The E_corr_ of TC4-p (-0.287 V) is slightly more positive compared to TC4 substrates (-0.304 V), suggesting that plasma treatment can improve the corrosion resistance of the substrate. The MAO coating of CaP-p, and Sr-CaP-p had higher E_corr_ (-0.109 and -0.026 V) compared to titanium alloy, suggesting improved corrosion resistance of TC4 resulting from MAO and plasma treatment. This improved corrosion resistance in the human body is essential for metal implant development.

**FIGURE 4:**
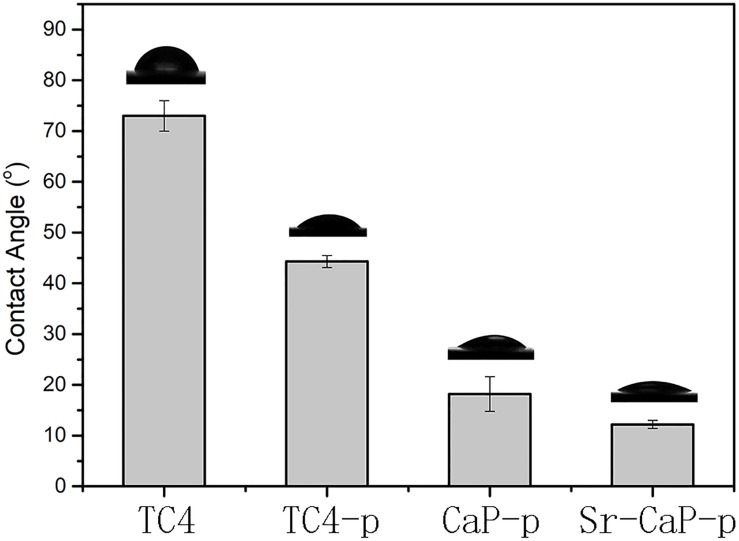
Contact angle measurement of water on the surface of different samples.

Hydrophilicity is critical for the biological activity of bone tissue implants (Albrektsson and Wennerberg, 2019). Knowing this, we measured the water contact angle just before cell incubation. The water contact angles on the surfaces of the TC4 and customized coatings are shown in Figure 4. The static contact angle of water droplets on the original, as-polished Ti disk was approximately 73.0 ± 3.0°. After plasma treatment, the water contact angle changed to 44.3 ± 1.2°, suggesting that the obtained coating had hydrophilic properties. After the MAO process, the water contact angles of CaP-p and Sr-CaP-p decreased to 18.2 ±3.4° and 12.2 ± 0.8°, respectively. The micro/nano hierarchical structure exhibited a powerful capillary function, which resulted in increased contact area and accelerated water droplet spread. These results suggest that the excellent wettability may be beneficial for protein adsorption, adhesion, and spreading of cells.

### Cell Proliferation and Cell Viability

**FIGURE 5:**
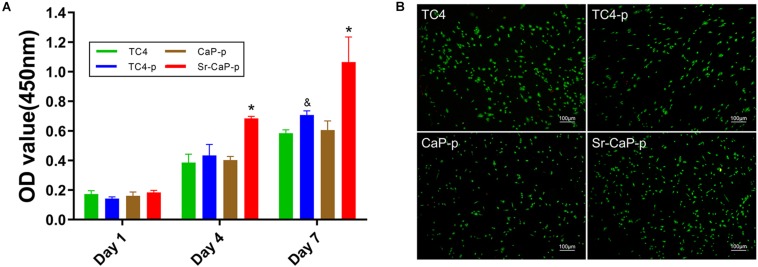
**(A)** Cell proliferation of rBMSCs on TC4, TC4-p, CaP-p, Sr-CaP-p at 1, 4, and 7 days. (* indicates statistical significance *p* < 0.05 vs. TC4-p and & indicates statistical significance *p* < 0.05 vs. TC4). **(B)** Cell viability of rBMSCs on different groups at 24 h. Live cells were stained with green dots and dead cells were stained with green dots. Scale: 100 μm.

Cell proliferation on days 1, 4, and 7 was measured for cells grown on TC4, TC4-p, CaP-p, and Sr-CaP-p (Figure 5A). All groups show a tendency to increase in cell mass, indicating that they provide the feasibility of suitable substrates of cell growth environment. On day 1, there was no difference in cell proliferation among the groups. While on day 4, the proliferation rate of the Sr-CaP-p group was significantly higher than those of the other three groups. Lastly, on day 7, the proliferation rate of the TC4-p group was significantly higher than that of the TC4 group, and the rate of the Sr-CaP-p group was significantly higher than that of theTC4-p group. These results demonstrate that Sr-CaP-p promotes the highest rate of cell proliferation. To evaluate the cytotoxicity of the coated samples, live-dead cell staining (red dots = dead cells; green dots = live cells) was performed. Cell viability was assessed using fluorescence microscopy (Figure 5B). All groups were observed under the same magnification. Samples tested from all four groups showed low cytotoxicity indicated by the low number of red dots (dead cells) observed.

### Cell Adhesion

**FIGURE 6:**
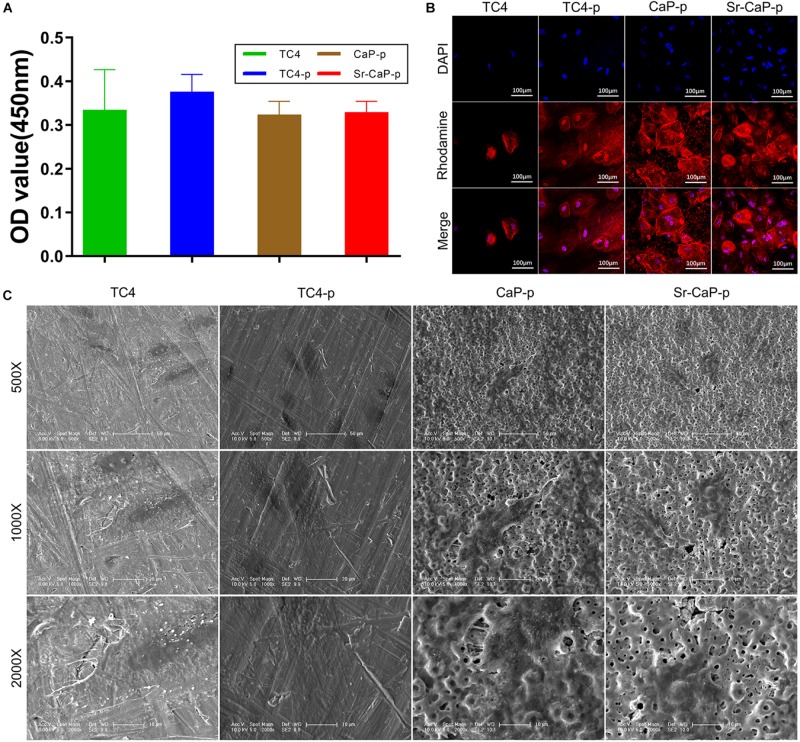
**(A)** Cell adhesion of rBMSCs on different groups at 2 h. **(B)** Confocal laser scanning microscopy images of rBMSCs on different groups at 2 h (red, rhodamine-phalloidin for F-actin; blue, DAPI for nucleus). Scale: 100 μm. **(C)** SEM micrographs of rBMSCs on different groups at 12 h. Scale: 50 μm (500 Scale), 20 μm (1000 Scale), 10 μm (2000×).

Short-term cell adhesion can be quantified by cell viability (Figure 6A). Cells were allowed to adhere for 2 h before quantification was performed (similar to a previous study, Zhang et al., 2018). The number of adherent cells on TC4-p was higher than those on the CaP-p and Sr-CaP-p groups, which had nearly identical OD values (which represent the relative cell number). Additionally, initial cell adhesion behavior was assessed by visualizing the rhodamine-labeled phalloidin-stained cytoskeleton, and DAPI-stained nuclei using confocal microscopy (Figure 6B). rBMSCs were shown to adhere to all the samples. Notably, cells adhered to the Sr-CaP-p had many intercellular connections, as well as clear and well-ordered cytoskeleton/microfilaments. Additionally, there was an obvious rearrangement of the cytoskeleton and good directionality.

To avoid the effect of fluorescent dye adsorption, showing the micropores clearer, we used a SEM to observe the passage of cells on the coating (Figure 6C). Morphologically, at higher magnification (1000X, 2000X), cells in the TC4 group had a flat structure and the intercellular connections between cells were almost invisible. Cells in the TC4-p group exhibited a typical fusiform shape with clear pseudopods, and intercellular filaments could clearly be seen. In the CaP-p group, a longer irregular fusiform structure was observed. The pseudopods are extended directionally to micropores. For the Sr-CaP-p group, there is an extended structure with some cell–cell interactions, and the extended pseudopods with multi-direction attach well to the micropores. These results show that TC4-p, CaP-p, and Sr-CaP-p all demonstrated better cell adhesion than TC4, while Sr-CaP-p has the highest performance.

### Osteogenic Differentiation

**FIGURE 7:**
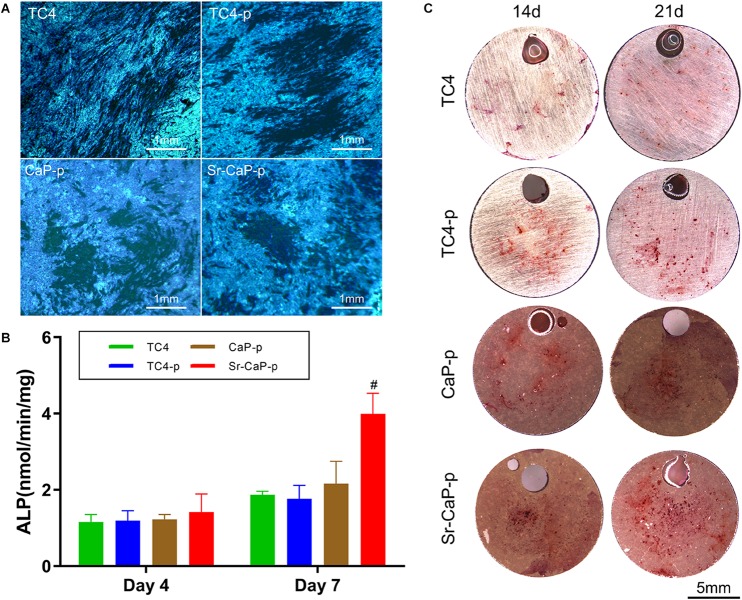
**(A)** Locally magnified picture of ALP staining images at 7 days. Scale: 1 mm. **(B)** ALP activity at 4 and 7 days (# indicates statistical significance *p* < 0.05 vs. CaP-p). **(C)** Alizarin red staining images of different groups at 14 days. Scale: 5 mm.

Alkaline phosphatase staining was performed to detect ALP expression. Cells seeded on TC4, TC4-p, CaP-p, and Sr-CaP-p were all stained (Figure 7A) and day 7 was chosen to represent early-stage differentiation. The nodules in the deep-stained area of TC4 were scattered, while those of the other three coating groups were closer. From the perspective of the denseness, nodules in CaP-p and Sr-CaP-p have deeper staining. Osteogenesis was accurately measured by quantifying the ALP activity (a measure of the differentiation level of osteoblasts). Early osteogenesis was measured at days 4 and 7 in quantification. As time progressed, the ALP activity expression increased (Figure 7B). At day 4 and 7, the highest ALP activity expression was observed in the Sr-CaP-p group. This result was consistent with the staining result (Figure 7A). Overall, the Sr-CaP-p group had the best ALP activity during early osteogenesis.

The formation of mineralized nodules is one of the most important phenomena at the end stage of osteoblast differentiation and represents the maturation of osteoblasts. To assay this, alizarin red staining is performed to measure the extent of mineralization (Gregory et al., 2004). Alizarin red staining was conducted on day 7 and 14 (Figure 7C). The number of stained red spots (representing calcium nodules) was used to quantify alizarin red staining. Generally, more calcified nodules are observed at the advanced stage (21 days) compared to the middle stage (14 days) in each group. Specifically, the number of calcified nodules in the TC4-p group on days 14 and 21 was higher compared to the TC4 group. On days 14 and 21, nodules in Sr-CaP-p were increased compared to the CaP-p group. Moreover, the Sr-CaP-p group had significantly more nodules on days 14 and 21 compared to every other group. We conclude that among the groups, Sr-CaP-p possessed the greatest osteogenesis.

## Discussion

Biomaterials with osteogenesis and enhanced biocompatibility are important for the production of orthopedic implants. In this study, we used SLM technology to prepare titanium alloys. SLM technology has a high degree of processing freedom (unlike traditional design) making it possible to produce medical devices of various shapes and structures. The basic principle of SLM technology is that metal powder is completely melted under the heat of laser beam, and formed after cooling and solidification. SLM can quickly prepare orthopedic implants. To further enhance the biocompatibility and osteogenesis of the orthopedic implant, suitable modification is necessary. In this study, we fabricated and characterized a coating treated with air-plasma and MAO. To investigate the biocompatibility and osteogenesis of the coating, rBMSCs were cultured on the coating surface and various assays were performed to determine the suitability of the coatings.

Biocompatibility and osteogenesis are effective evaluation methods for orthopedic implants (Constantin et al., 2019). When assessing the biocompatibility of a biomaterial, cytotoxicity is an important early measure that can be used to qualitatively and quantitatively determine the potential biological risks. Cell adhesion and cell proliferation are also effective for the determination of a material’s biocompatibility. At the cellular level, assessment of osteogenic capacity can be performed using both ALP and alizarin red staining. This staining demonstrates some of the key proteins in the stem cell osteogenesis signaling pathway (Aminian et al., 2016).

The suitability of air-plasma-treated titanium alloy for stem cell culture has not been directly tested; therefore, we first performed a biocompatibility verification on a single air-plasma-treated titanium alloy. Cell proliferation, cell adhesion, and cell viability experiments demonstrated faster cell growth, more extended cytoskeleton, clearer cell morphology, and lower cytotoxicity on TC4-p compared to TC4. Specifically, there was a significant difference in cell proliferation on day 7. Spreading cell morphology was clearly observed in cells grown on the TC4-p coating which aids in promoting cluster growth and proliferation of rBMSCs. Although ALP activity of TC4-p is not significantly higher than that of TC4, the results of ALP and alizarin red staining (as shown in Figures 7A,C) show that single air-plasma coating enhances the osteogenic activity. This evidence demonstrates that air-plasma treatment can significantly improve the biocompatibility of inorganic implants and may be conducive for osteogenesis. After air-plasma treatment, hydroxyl radicals are added to the treated surface (Tsougeni et al., 2015; Kurzyp et al., 2017; Sathish et al., 2019; Wieland et al., 2020; Zhang et al., 2020), further reducing water contact angle shown in Figure 4, which forms an adsorption effect with adhesion proteins and enhances biocompatibility. Hydrophilicity is critical for the bioactivity of bone tissue implants and can improve cell adhesion and proliferation. It has been reported that the surface energy and hydrophilicity would affect the biocompatibility positively by changing the adsorption capability of the surface (Fan and Guo, 2020). Cell adhesion, migration, and growth are related to the degree of roughness and hydrophilicity of the biomaterial’s surface. These properties may explain the improved biocompatibility of TC4-p. When hydroxyl radicals are formed on the treated surface, the OH^–^ attracts Ca^2+^ ions to the film surface and increases the calcium ions concentration on the surface of the TC4-p sample. As the calcium ions accumulate, then combine the PO_4_^3–^ in the DMEM to form CaP apatite. Thus, the CaP apatite formed on the TC4-p sample may improve the osteogenic activity (Huang et al., 2015). Since both TC4-p and CaP-p groups promote osteogenesis via the osteoinduction effect of the CaP, they have a similar performance on the *in vitro* osteogenesis experiment, especially the mineralization of extracellular matrix (Figure 7C). Osteogenesis is enhanced in both the TC4-p and CaP-p groups. However, the enhancement of osteogenesis by air-plasma treatment is limited, and it is necessary to introduce a coating that is beneficial to osteogenesis.

To further enhance the osteogenesis of the implant material, active ion-doping can be performed. Strontium has a long history of being utilized as an active component of orthopedic implants and bone tissue engineering materials (Pilmane et al., 2017). Whether as a substrate or modification, strontium has excellent osteogenesis. In this work, the Sr-CaP-p group enhanced osteogenic activity better than any other group, which indicates a beneficial role for Sr. Our previous studies have also shown that Sr ion-exchange zeolite-A coatings can remarkably enhance the osteoinductive ability of porous titanium alloys (Wang et al., 2018). Peng et al. (2009) also found that Sr promoted osteogenic differentiation of MSC by activating Ras/MAPK signaling pathway and downstream transcription factor Runx2. Overall, we found that the use of strontium in the coating could enhance the osteogenesis of the implant.

The comparison of TC4-p and TC4 confirms that air-plasma treatment enhances biocompatibility. To improve osteogenesis, CaP was chosen to be an incorporation coating for strontium. Strontium-doped or -undoped CaP was combined with air-plasma treatment to study the induction of osteogenesis. With regard to biocompatibility, the cell proliferation results showed excellent cell growth capability on Sr-CaP-p. It is worth noting that the enhancement of cell proliferation in the CaP-p group is not significant, which is consistent with some reports (Klymov et al., 2016; Nguyen et al., 2019). Confocal microscopy images showed that cells in the CaP-p group spread well with clear microfilaments while cells in the Sr-CaP-p group had a spindle-shaped cell morphology with actively-dividing cells. Electron micrographs of the two groups of cells show obvious pseudopods that extended to the micropores, which were not observed in the TC4 and TC4-p groups. This is due to the micro-scale pores formed on the surface by the MAO method which is conducive to cell attachment and provides a suitable microenvironment for early bone repair as previously reported (Alves et al., 2019). In terms of osteogenesis, both ALP activity (Figure 7B) and alizarin red staining (Figure 7C) show that Sr-CaP-p has the best osteogenesis at both the early and advanced stages. A comprehensive comparison of each group shows that the biocompatibility and osteogenesis of Sr-CaP-p are significantly improved. In summary, air-plasma treatment introduces a large number of hydroxyl groups and reduces the water contact angle, thus enhancing protein adsorption and promoting stable protein release. Additionally, Sr-CaP coating potentially works in concert with air-plasma treatment to improve biocompatibility and induction of osteogenesis.

## Conclusion

In this study, we demonstrated the biocompatibility and osteogenesis of air-plasma-treated strontium-doped phosphate coating. It had been successfully developed and characterized after applying MAO and air-plasma treatment on SLM-produced titanium alloys. Air-plasma treatment does not change the structure and composition of materials but it improves the wettability and corrosion resistance of the substrate. To investigate the biocompatibility and osteogenesis of TC4-p and Sr-CaP-p, various assays measuring cell proliferation, cell adhesion morphology and quantification, cell viability, ALP staining and quantification, and alizarin red staining were performed. Overall, rBMSCs adhered, proliferated, and induced osteogenesis throughout the culture period. Pure air-plasma treatment enhanced biocompatibility and slightly promoted osteogenesis. The results also showed that strontium-doped phosphate coating significantly enhanced both biocompatibility and osteogenesis. Therefore, these results show that (i) air-plasma treatment can be an efficient method to enhance the biocompatibility of a titanium alloy; and (ii) manufactured Sr-CaP-p can be a beneficial modification to enhance the biocompatibility and osteogenesis of a titanium alloy.

## Data Availability Statement

All datasets generated for this study are included in the article/Supplementary Material.

## Author Contributions

HX and RL collected the experimental data, performed data analysis, and wrote the manuscript. YW and BY performed a part of data analysis. DL and YQ oversaw the project and performed the overall editing of the manuscript.

## Conflict of Interest

The authors declare that the research was conducted in the absence of any commercial or financial relationships that could be construed as a potential conflict of interest.
