# Supplementary material for: Improved Osteogenesis of Selective-Laser-Melted Titanium Alloy by Coating Strontium-Doped Phosphate With High-Efficiency Air-Plasma Treatment
Source: Front Bioeng Biotechnol. 2020 May 12;8:367. doi: 10.3389/fbioe.2020.00367 (PMC7235326; doi:10.3389/fbioe.2020.00367)
Supplement: Supplementary file 1 [file Image_1.pdf]

Supplementary Material

Supplementary Figures

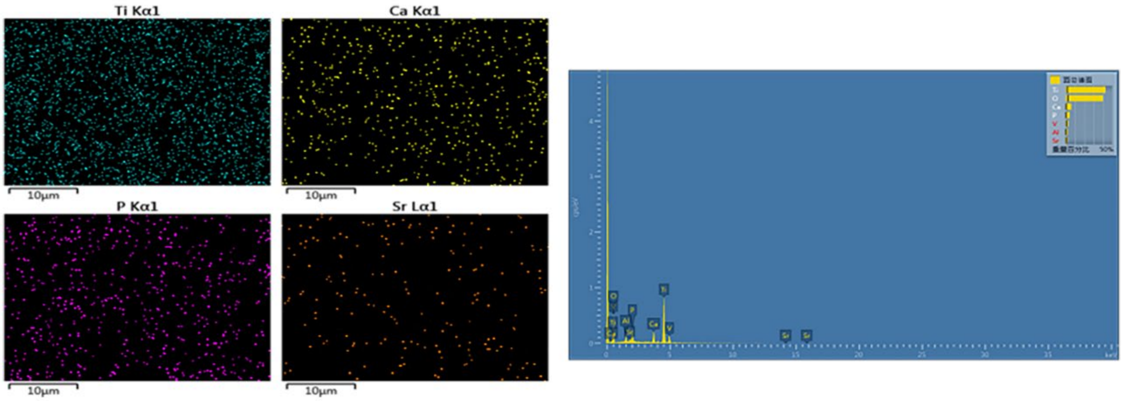

Figure S1. EDS mapping of the distribution of Ti, Sr, Ca, and P.
